# *"Treatment is of primary importance*, *and social assistance is secondary"*: A qualitative study on the organisation of tuberculosis (TB) care and patients' experience of starting and staying on TB treatment in Riga, Latvia

**DOI:** 10.1371/journal.pone.0203937

**Published:** 2018-10-17

**Authors:** Karina Kielmann, Nicole Vidal, Vija Riekstina, Maria Krutikov, Marieke J. van der Werf, Evita Biraua, Predrag Duric, David A. J. Moore

**Affiliations:** 1 Institute for Global Health and Development, Queen Margaret University, Edinburgh, United Kingdom; 2 Centre for Tuberculosis and Lung Disease, Riga East University Hospital, Riga, Latvia; 3 University of Latvia, Faculty of Medicine, Internal Medicine Department, Riga, Latvia; 4 TB Centre, London School of Hygiene and Tropical Medicine, London, United Kingdom; 5 European Centre for Disease Prevention and Control, Stockholm, Sweden; Maastricht University, NETHERLANDS

## Abstract

**Background:**

Vulnerable individuals with tuberculosis (TB) struggle to access and stay on treatment. While patient-related and social barriers to TB treatment adherence are well documented, less is known about how the organisation and delivery of TB care influences adherence behaviour.

**Aim:**

To examine the influence of TB service organisation and culture on patients’ experience of starting and staying on treatment in Riga, Latvia.

**Methods:**

An intervention package to support adherence to TB treatment amongst vulnerable patients in Riga, Latvia was piloted between August 2016 and March 2017. Qualitative observations (5), interviews with staff (20) and with TB patients (10) were conducted mid-way and at the end of the intervention to understand perceptions, processes, and experiences of TB care.

**Results:**

The organisation of TB services is strongly influenced by a divide between medical and social aspects of TB care. Communication and care practices are geared towards addressing individual risk factors for non-adherence rather than the structural vulnerabilities that patients experience in accessing care. Support for vulnerable patients is limited because of standardised programmatic approaches, resource constraints and restricted job descriptions for non-medical staff.

**Conclusion:**

Providing support for vulnerable patients is challenged in this setting by the strict division between medical and social aspects of TB care, and the organisational focus on patient-related rather than systems-related barriers to access and adherence. Potential systems interventions include the introduction of multi-disciplinary approaches and teams in TB care, strengthening patient literacy at the point of treatment initiation, as well as stronger linkages with social care organisations.

## 1 Introduction

The Baltic republic of Latvia is one of 18 high priority countries for tuberculosis (TB) control in the World Health Organization (WHO) European Region, and has a high burden of multidrug-resistant tuberculosis (MDR-TB) [1,2]. In 2016, 560 new TB cases and 100 retreatment cases were registered in Latvia, among them 32 and 21 MDR TB cases respectively [3]. The country has undergone drastic changes in TB notification rates over the past 20 years [2]. After the collapse of the Soviet Union in the early 1990s, TB rates increased substantially, and only started to decline after 1999, partly due to rapid economic growth in the Baltic region and the adoption of a centralised, strictly followed programmatic approach to management of TB. The TB incidence rate increased again in 2012, coinciding with the global economic crisis that hit Latvia harder than any other EU Member State. Unemployment rates increased, and a large part of the population experienced a decline in socio-economic status and health [4]. At the same time, the number of HIV cases in Latvia rose sharply; by 2012, 20% of those diagnosed with TB also had HIV.

From 2013 to 2016, the situation improved again, with registered new TB cases declining from 38.1 per 100 000 in 2013 to 28.4 in 2016 [5]. However, vulnerable and socially marginalized individuals are disproportionately affected [6]. Despite a high treatment success rate (88%) among new drug-susceptible TB patients, factors influencing unsuccessful treatment outcomes in vulnerable individuals including unemployment, alcohol and drug use, and HIV are consistently reported in epidemiological studies on TB and drug-resistant (DR) TB in the Baltic States [1]. However, there are no known studies that look at the responsiveness of the health system vis-à-vis vulnerable individuals, for whom a number of these risk factors may be conflated. Here, we draw on a qualitative study of TB care in Riga, Latvia, to examine how patients with atypical circumstances that compromise their health and health-seeking navigate standardised TB care pathways.

Social science literature on infectious disease control programmes suggest that the way in which the disease, treatment approach, and treatment adherence are conceptualized have important bearing on how care is delivered. Although there are recent appeals for a patient-centred focus in tuberculosis care [7], this is a relatively novel paradigm for TB. Unlike HIV care, which espoused patient-centred approaches to address behavioural risk factors from the outset, TB management has historically focused more tightly on biomedical determinants of infection [8]. The predominant perspective, from which directly observed therapy (DOT) and other clinic-based interventions are designed, views the patient as a passive recipient of professional medical care [9]. Though previously regarded as a vital element of global TB control programmes, DOT has been criticized as paternalistic, and when rigidly implemented, serves to “*reinforce asymmetrical relations of power between different constituencies*, *and to strengthen conventional modes of provider-patient interaction”* [10]. Advocates of a patient-centred approach to TB care have been vocal about the need to change terms commonly adopted by TB programmes, such as terms like ‘compliance’, ‘defaulter’, and ‘control’ which reflect top-down and disempowering views of patients [11]. However, the change in language from ‘compliance’ to ‘adherence’ which ostensibly grants greater agency and choice to patients may in fact place undue responsibility for treatment completion on the patient [12] and detract from the structural factors affecting an individual’s ability or will to complete treatment [13]. An emphasis on structural vulnerabilities recognises that some patients struggling to stay on treatment live within risk environments [14] where a range of social determinants of ill health including gender, poverty, and social marginalisation diminish their capacity for agency in health-seeking behaviour, including following a treatment regimen [15–17].

Patient-centred approaches that take account of underlying social and structural factors influencing patients’ abilities to begin and stay on treatment [18–20] may extend beyond the clinic to include families, peers, and social networks [21] but must also include critical reflection on the ways in which health systems themselves may perpetuate a paternalistic and disempowering view of patients. In this paper, we argue that it is important to understand the clinic as a site of social and professional norms and relationships, in line with recent calls for actor-oriented and relational research on health systems [22]. Examining the organisational context and social relations of TB care can shed light on health provider assumptions of what constitutes ‘good care’ and how it should be delivered. In turn, these insights reveal opportunities to enhance patients’ experience of TB care within the health system, from coming to terms with a diagnosis, to navigating complex pathways to care, and staying motivated while on treatment.

## 2 Methods

### 2.1 Context of study

This paper reports qualitative findings drawn from a mixed-methods process evaluation of a pilot intervention study that took place between January 2016 and March 2017 in Riga, Latvia under the auspices of the Centre for Tuberculosis and Lung Diseases (CTLD). The CTLD is one of six clinical centres of the Riga East Clinical University Hospital (Riga, Latvia), with TB diagnostic and treatment services free of charge and financed by the state. Patients are usually diagnosed and initiate treatment in hospital and continue on ambulatory basis once they are smear negative, able to tolerate treatment well and when it is possible to ensure ambulatory directly observed therapy (DOT). Culture positive samples from each patient undergo drug susceptibility testing. All patients are offered HIV testing; those found to be HIV infected are offered co-trimoxazole preventive therapy (CPT) and antiretroviral therapy (ART) [1]. In recent years, the TB treatment success rates in Riga and the Riga region have been high overall (80.5% in 2014) but remain below the target rate of > 85%. The current model does not work for some patients, as indicated through the loss-to-follow-up rates of approximately nine percent of all patients (personal communication, TB doctor, CTLD), indicating that there are some persistent challenges with adherence to TB treatment.

We piloted an intervention designed to improve adherence of patients to TB treatment. There were three components to the intervention. Firstly, a two-day training workshop was held to strengthen patient communication skills among all staff involved with TB patient care. Secondly, a psychosocial risk screening tool to identify those TB patients likely to struggle with adherence to treatment was developed, based on an existing adherence risk screening tool developed for TB services in London in 2012. It was adapted to the Latvian context and streamlined to facilitate incorporation into routine clinical practice. Thirdly, patients identified by the screening tool as having one or more risk factors for non-adherence were provided with an additional adherence support meeting with the head ambulatory nurse. These patients were closely followed up and offered help with finding suitable housing, referral to a psychologist and financial support to cover travel costs, where needed. The objectives of the pilot intervention study were: 1) to compare adherence and culture conversion times in patient cohorts before and after implementation of the intervention; and 2) to conduct a process evaluation of implementation of the intervention, considering patient and staff perceptions and experiences of delivery of care during the intervention period. In this paper, we draw on the qualitative data collected in the process evaluation to highlight the importance of social organisation and culture of TB service delivery in the trajectories of vulnerable patients on treatment.

### 2.2 Sampling and recruitment procedures

The European Centres for Disease Control (ECDC) issued invitations to a wide range of European institutions providing specialised TB care to act as host sites for implementation research to contribute to the goal of “increased TB treatment adherence and improved treatment outcomes among specific hard-to-reach and vulnerable population groups in the EU/EEA”. The chief of medicine at the CTLD expressed interest to host a project and sent an official agreement letter to ECDC, granting permission and arranging the logistics of data collection for the researchers from LSHTM and QMU during two separate periods of fieldwork at CTLD and its affiliated partner and satellite sites. Participants for the qualitative interviews were purposively selected based on their involvement in either providing or receiving TB care. Fourteen health providers involved in TB care and support were interviewed. They were either based at CTLD or were from relevant referring departments and affiliated institutions including: the ‘satellite’ DOT clinic in the city centre, the MDR-TB ward of CTLD’s inpatient department, a shelter that refers individuals to CTLD for TB testing, and a non-governmental organisation (NGO) providing support and counselling services for individuals affected or co-infected with HIV.

At the time that patient interviews were held (four months into the intervention), 30 patients had initiated TB treatment. On screening, about half of these patients exhibited one or more risk factors for poor adherence to treatment. The TB nurse at CTLD approached each of these patients regarding their willingness to participate in the study, and ten patients expressed interest. After explaining the project to the patients, written consent was obtained for the researchers to conduct and record the interview using a digital voice recorder.

### 2.3 Data collection

Data collection took place over the course of four site visits between January 2016 and March 2017 and included observations, semi-structured interviews, and review of patient records and notes. NV and KK conducted semi-structured interviews and observations with staff and patients midway and at the end of the intervention period, with the help of two Latvian research assistants (RAs) who provided ongoing translation. During the first round of data collection, all 14 health providers and 10 patients were interviewed. Follow-up interviews were conducted with six of the 14 health providers during the second round of data collection. To preserve anonymity in reporting, all patients and staff members interviewed were assigned culturally appropriate pseudonyms.

Patient interviews (Table 1) were conducted in Latvian, Russian or English and elicited information about patients’ backgrounds, their family situation, and their health, including being diagnosed with TB. We then focused on their experiences with treatment initiation, care and support, communication with providers at each stage of treatment, as well as the broader familial and social context of their medicine-taking behaviour. We gained further information on these patients’ trajectories within the health system from notes on patients’ records that were reviewed with the assistance of the CTLD head nurse to supplement the development of patient case studies. These patient records were a subset of a larger sample of records included in a retrospective record review that was conducted to collect quantitative data on treatment adherence and on bacteriological conversion times (not reported here).

**Table 1:** Patient interview participants.

Pseudonym	Characteristics
1. Jevgenijs	Male, early 50s, originally from Ukraine, unstable housing status, history of alcohol and substance use
2. Sergejs	Male, approx. 60s, from Riga, receives disability support, has heart-related health problems, lives alone
3. Viktors	Male, mid 30s, Russian speaker, bartender, history of substance use
4. Andris	Male, early 50s, part-time mechanic, mobile home, separated from spouse and children.
5. Igors*	Male, mid 30s, unemployed, from Riga, wife and child in treatment for TB, history of alcohol use
6. Amadi	Male, early 30s, refugee from Eritrea, was living in refugee camp
7. Dainis	Male, late 40s unemployed, from Riga, second time being treated for TB, history of alcohol use
8. Ludmila	Female, approx. mid-40s, unemployed, from Riga but declared in another city, history of alcohol use and victim of domestic violence
9. Kaspars	Male, approx. 40s, unstable employment, has lived in Riga for 20 years but originally from another region in Latvia
10. Natalija*	Female, approx. mid-30s, Russian speaker from Riga, lives with husband and child

*Not referred to in manuscript.

Staff interviews (Table 2), conducted in Latvian and English, initially elicited information about staff members’ background, length of time working at CTLD, their roles, and their particular responsibilities in TB care. We then moved to asking about the organisation and processes of TB care, and obstacles and facilitators to delivery of the intervention within this setup. Staff members were encouraged to focus on concrete examples of patients (without naming them) in order to illustrate their perspectives on the challenges of adherence to TB treatment for this population. Structured observations were conducted midway and at the end of the study period at different points along the patient pathway including: the CTLD DOT room, the TB physician’s office, the CTLD registration desk and the satellite DOT clinic in Riga’s city centre.

**Table 2:** Staff interview participants.

Pseudonym	Staff member role	Follow up interview conducted?
1. Marta*	Sputum collection nurse, CTLD	
2. Dr L*	Head of CTLD ambulatory department	
3. Dr A	TB physician 1, CTLD ambulatory department	x
4. Dr B	TB physician 2, CTLD ambulatory department	x
5. Guna	TB nurse, CTLD ambulatory department	x
6. Dr C	TB physician 3, MDR-TB ward)	x
7. Jana*	Head TB nurse, MDR-TB ward	
8. Marija	TB nurse, MDR-TB ward	
9. Alise*	Psychologist/social worker, HIV NGO	
10. Sofija*	Social worker, homeless shelter	
11. Anna	Courier, CTLD ambulatory department	x
12. Anita	Head TB nurse, CTLD ambulatory department	x
13. Ilze	Social worker, CTLD	
14.Kristine	TB DOT nurse, satellite clinic	

*Not referred to in manuscript.

### 2.4 Data management, processing and analysis procedures

As interviews were conducted with the help of an RA, transcripts of recordings were not verbatim, but of the English translation of responses. The RA listened to each recording, checking over and editing the transcripts where needed. NV and KK reviewed all transcripts (interviews, observations and field notes) several times before entering them into NVivo qualitative data analysis software, version 10. A process of open coding around the broad topic of ‘organisation of TB care for vulnerable patients’ led to a framework focusing on four different dimensions of care: infrastructural context; professional roles and relationships; procedures and processes; and patient-provider interaction and communication. In further analysis, we examined how these dimensions played out at different stages of standardised processes including risk screening, diagnosis and treatment initiation, DOT and adherence support.

### 2.5 Ethical considerations

Ethical approval was obtained by the ethical review boards at Queen Margaret University Edinburgh (UK), The London School of Hygiene and Tropical Medicine (UK) and the Riga Stradins University, Latvia. All identifying information relating to staff and patients was anonymised. In the paper, we used pseudonyms rather than numbers to humanize patients’ and staff narrative accounts and quotes. For some staff interviewees, we used initials that have no connection with actual names. All participants were provided with information sheets detailing the objectives of the study and their rights as participants. The researchers reviewed the information sheet with the participants prior to the start of each interview and written informed consent was obtained from each study participant.

## 3 Results

In this section, we present results from the qualitative components of the process evaluation under four sections that correspond to significant points along patients’ trajectories in TB care: risk screening, diagnosis and treatment initiation, adherence support, and DOT. In documenting these four phases of patient pathways through the clinic, we focus on organisational context and culture of TB care as important systems features that influence patients’ experience of starting and staying on treatment.

### 3.1 ‘*It’s very difficult to define this vulnerability’*: from risk factors to structural vulnerabilities

A central feature of epidemiology is the investigation of individual and population-level risk factors as primary causes of disease or behaviours deemed to be detrimental to health. The risk factor approach has been criticised as overly individualistic and reductionist as it masks the underlying reasons that place particular individuals at risk [23]. Despite considerable debate around its limitations, the emphasis on ‘risk factors’ continues to dominate mainstream epidemiological research. In clinical practice, this approach is reflected in screening tools that assist triage and decision-making around eligibility for further diagnostic procedures, treatment regimens or other forms of care and support.

For staff at CTLD, screening of patients at the time of treatment initiation is used to assist in early identification of risk factors for poor adherence to treatment. By early March 2017, 67 patients had been enrolled at CTLD, and just over half of them (52.2%) were noted to have one or more risk factors for poor adherence to TB treatment, the most commonly noted being: excessive alcohol consumption (n = 14); living outside of Riga city or not being a registered resident of Riga (n = 12), and social isolation (n = 14), defined as limited or no recourse to family or friends who could support them whilst on treatment.

Through a checklist, the screening tool enables the TB nurse to take note of specific issues that hinder a patient’s capacity to follow a treatment regimen. However, the standardised nature of the tool isolates specific risk factors as generic patient-related characteristics, masking the complex dynamics of underlying vulnerability that puts some patients not only at risk for ‘poor adherence’ to TB treatment, but more broadly, at risk for compromised physical and mental health.

Being ‘at risk’ for poor adherence to treatment starts long before the diagnosis of TB and has more to do with situational circumstances than with individual characteristics. For a number of patients interviewed, the entry point to TB care was not directly related to TB symptoms but to other illnesses or conditions that compounded the burden of physical disability and distress and complicated access to care. Ludmila had been on treatment for two months when we spoke to her and was noted to have two main risk factors for poor adherence: a history of alcohol abuse and social isolation. However, of equal importance to her ‘risk’ profile is the fact that Ludmila is diabetic. When she sought care for a cough and raised temperature that would not disappear, she was told that her diabetes had weakened her immune system. Upon being informed that she had TB following an x-ray, she expressed shock and uncertainty about how to manage the dual burden of disease. Similarly, Jevgenijs, who was initially admitted with an injury incurred while drunk, has also had problems with alcohol and narcotics use in the past which puts him ‘at risk’ of poor adherence. The diagnosis of TB places an additional burden on his pre-existing HIV status which he lamented as “*creating many problems in my life*”. Illness histories of patients deemed ‘at risk’ of poor adherence were often closely intertwined with precarious working conditions. Like Jevgenijs, Andris and Dainis were diagnosed with TB through a circuitous route. Both suffered accidents while working, which led them to seek acute care. Andris, a part-time mechanic in his early 50s, broke a bone in his upper shoulder. Dainis, a stair cleaner in his late 40s, suffered a bad fall a number of years ago and broke his ribs which led to him not being able to work.

Jevgenijs and Andris were also deemed at risk for poor adherence to treatment because they had no fixed abode in Riga. Andris is originally from a town about one hour away. Separated from his wife, he lives in a camper van. In his profile, as in that of others we interviewed, the significance of being ‘without fixed abode’ extended far beyond a bureaucratic hurdle in accessing municipal services to encompass the consequences of mobility, fragmented social ties, marginalisation, and interrupted patterns of care-seeking. When Andris had his accident, he first went to a doctor in Riga, but was advised to seek help from a doctor in his hometown. The doctor in his hometown suggested he go for another x-ray in Riga, which revealed “*something wrong with my lungs*”. He was admitted to the hospital and discharged after a week but remained unsure about whether he had TB or not. Kaspars, originally from a town approximately 200 km away, has been in Riga for twenty years, yet experiences acute job insecurity. Since being forced to give up his small business during the 2008 financial crisis, he has been ‘unofficially’ employed in construction work. Anxious about missing sporadic work opportunities, he told us that he generally avoided doctors and self-medicated in the case of feeling unwell. In his case, he delayed seeking care for the fever and unusual pain in his side that he was experiencing, only calling emergency services when the pain became severe.

The impact of having ‘no fixed abode’ is especially poignant for migrants. Amadi, a refugee in his 30s from Eritrea, came to Latvia in July 2016 via a long and difficult route with ‘transit’ time spent in a number of countries as an asylum seeker. He was tested for, and diagnosed with, TB as part of the requirements of his refugee status. In addition to TB, he was also found to have nutritional deficiencies and pleurisy. He was hospitalised for a few weeks, then discharged, but returned to see the doctor again after feeling unwell, and went back to the hospital, this time for two months. At the time of interview, he was living in a refugee camp outside of Riga Municipality and was not working. He speaks English but does not speak Latvian or Russian; in his interview with us, he became increasingly despondent and pessimistic about his ability to negotiate not only the health services, but more broadly the ‘system’ per se.

Among the CTLD staff, there was consensus that the discreet ‘risk factors’ identified through the screening checklist predisposed individuals to non-adherence, but also shared agreement that the ‘*social part’* was as relevant to the progress of patients on TB treatment as their clinical profile. Some staff recognised, that ‘risk factors’ were intertwined and that individuals ‘at risk’ of poor adherence were embedded in social contexts that were characterised by their vulnerable structural position. Dr C, who works in the MDR-TB ward, provided an example of the challenge in assessing who was really ‘at risk’ of poor adherence:

*It’s very difficult to define this vulnerability*. *For example*, *for some women we know that they probably have some money and* [health] *insurance and so on … but we don’t know what happens in their family*. *Maybe there is some violence in the family and this is the factor that impacts later on the adherence*.

In eliciting information about patients’ backgrounds, we found that a risk factor approach was inadequate, barely skimming the surface of the profound social challenges that many patients in this setting faced. For Ludmila and other patients introduced above, the circumstances leading up to a diagnosis of TB illustrate that individual ‘risk factors’ are often intertwined in a web of structural vulnerabilities. Individuals who experience compromised health, homelessness, and social and economic marginality have limited power and social capital to negotiate the health system, not only at the point of accessing a diagnosis, but throughout the treatment pathway, as we observe in the following sections.

### 3.2 ‘*In the hospital*, *it was like a factory’*: coming to terms with a diagnosis

Diagnosis is a critical transitional moment in the care-seeking trajectory of individuals. Through a diagnosis, signs are converted into symptoms, the individual becomes a patient, and the therapeutic course of action is legitimised through recourse to institutional knowledge and authority. In practice, however, diagnostic uncertainties abound, in particular for more complex and chronic diseases, which can involve navigating different levels and components of a health system. In this study, the system for managing ‘difficult’ cases of TB is well-laid out and effectively coordinated from the providers’ point of view, however patients who participated in the study all experienced some uncertainty and confusion during their diagnostic journey.

All patients with a confirmed TB diagnosis receive TB treatment in hospital for a minimum of 2 weeks while they are smear-positive, or longer, depending on their tolerance to medications, and social factors, including financial, housing, and general support circumstances. If found to have DR-TB, they are kept in an isolation ward until smear conversion. For a number of patients, the experience of initiating treatment during hospitalisation was stressful. Dainis, who initially sought care for broken ribs, described his initial transfer to the hospital as a “*big mess*”. He moved from the 4^th^ floor, where patients are admitted after the initial diagnosis, to the 7^th^ floor when they realised he had DR-TB. He was found to be resistant to one drug and then moved to the 6^th^ floor, familiar to him from his past admission for TB, 20 years ago. In contrast to the first time he had TB, there was no long conversation at this point; he was simply given documents to sign. Jevgenijs’ experience of diagnosis was also distressing. He was first referred to the infectious diseases department by his HIV doctor because of a high temperature and kept there for a week before being checked for TB. He was then sent to the TB hospital and admitted to the isolation ward where he was diagnosed with MDR-TB. He tried to escape but broke his hip and was re-admitted and placed in isolation.

During hospitalisation, the doctor who sees the patient first confirms the diagnosis, tells patients about the treatment regimen, potential side effects of medication as well as co-morbidities. This technical information is difficult to absorb at a time when patients are in a state of shock about their diagnosis. Anita, the head TB nurse at CTLD, noted that patients were often “…*a little bit afraid of the doctor* […] *it’s something* [about the] *authority as a doctor…because the doctor is so busy and* [they] *can’t ask them anything*”. Marija, a nurse on the MDR-TB ward of the main hospital was frequently asked to confirm what the doctor had said, or to provide further information, because patients had not digested the information the first time it was given to them. There was, she said, little room for patient education at this stage, as her main duty was to ensure distribution of the drugs and necessary injections.

Kaspars, who delayed seeking care, expressed anger with the delays in establishing a diagnosis: he underwent a number of tests and investigations in the infectious diseases ward before being moved to the TB hospital for further tests, where the doctor told him that the diagnosis was a big “*mystery*”. The uncertainty around the diagnosis was distressing—“*I didn’t sleep for 3 days*”–and when finally confirmed, disclosure occurred in an abrupt manner, with a doctor informing him in the presence of other patients. He felt that his rights to confidentiality had been breached and described the TB hospital as a “*factory*” where “*nobody takes care*, *nobody pays attention*. *Nobody*. *One patient*, *then the next*, *the next*…”

The hospital environment can be alienating not only in terms of the lack of communication, but also because it represents a space where normal social relations are suspended. For patients like Kaspars and Viktors, a bartender in his mid-30s, the hospital stay was sobering as they found themselves in the company of people they perceived as socially inferior to them. Kaspars noted the number of homeless people in the hospital, commenting that this might be the reason the doctors there did not seem to connect with their patients. Viktors, although frank about his own history of alcohol addiction and drug use, bitterly described his shock at being amongst “…*bums and addicts”*. He felt let down by a system that put him back into a context and an identity he was trying to escape:

*I haven’t drunk alcohol in 4 years, haven’t been out in a year. My one bad habit is smoking. I try to keep away from these people as much as possible. Now I am amongst them. I spent one week re-evaluating my whole life*.

Ludmila, who spent a month in the hospital, was more forgiving of her time as an inpatient: “*I have nothing bad to say about the doctors and the workers*. *They treated me so well*. *They have such a low salary and work in such a place*, *we can only thank them*”.

The time spent in the hospital represents an important starting point for patients as they come to terms with diagnosis. However, although she expresses gratitude for the care she received, Ludmila’s words reflect the extent to which the hospital is perceived as a de-humanising environment for patients and staff alike, and a missed opportunity for strengthening treatment literacy. During this critical phase when patients are initiated on to treatment, negative experiences are likely to have bearing on individuals’ self-identification as TB patients, their awareness of the course of illness and their readiness to embark on a long-term, challenging treatment regimen.

### 3.3 ‘*Treatment is of primary importance, and social assistance is secondary*’: Dividing the labour of TB care

Following their discharge from the hospital, patients are registered for ambulatory treatment. The ambulatory phase of treatment is strictly monitored, and within a context of programmatic vigilance, staff become more acutely aware of the factors that are likely to affect patients’ adherence patterns. From this point on, professional boundaries resulting in divisions in the organisation of TB care become more apparent, with implications for the allocation of resources and delivery of services that support patients on treatment.

During the initial consultation, the ambulatory clinic doctor takes a clinical history and establishes the treatment plan, while a TB nurse elicits patients’ address, contacts and phone numbers, as well as basic risk factors. Patients coming from the hospital are asked to sign a consent form to declare that they have understood the information provided regarding the duration of the treatment, what kind of complications are possible, and what happens if the patient stops taking the drugs. As in the hospital, the focus is on clinical management and the verbal exchange between the health staff and the patient is restricted to establishing risk factors and securing patient compliance to the treatment regimen. Dr A, one of the ambulatory doctors, affirmed the division of labour between herself and the nurse:

*I personally do not go very deep into the social problems because after the interview I ask* [Anita, head TB nurse] *how she feels about this person*. *For me as a doctor*, *I explain what the patient is to receive*, *for how long… the medical information*. *Then for me it’s interesting when* [Anita] *reports back to me because I don’t* [have to] *ask this…I appreciate this*.”

Following the consultation with the physician in the ambulatory clinic, all patients enrolled during the project period have an additional meeting with Anita. In this meeting, she elicits more information about the patients’ social circumstances, and spends time gauging their understanding of the condition and the course of treatment.

Overall, the experience of the initial consultations with the doctor and nurse were experienced by patients as positive and helpful, often contrasted with the confusion and distress experienced at time of diagnosis. Anita recalled her first meeting with Kaspars as terse—he appeared to have some “*mental problems*” and nervous mannerisms—but noted he had progressively gained trust and was able to take his treatment regularly. He contrasted his ambulatory care favourably with the negative experience of the hospital: “*everything here* [at CTLD] *is humane… it’s civilised*”. Dainis, too, praised the nurses, saying that they always showed interest in how he felt.

During her meeting with patients after their transfer to the ambulatory clinic, Anita fills out the psychosocial risk screening form to establish if the patient might require additional social support. Social support is categorised broadly into financial assistance, psychological support, or referral to a *narcologist*, a Soviet-era specialist psychiatrist trained in the study and treatment of alcohol and drug abuse. While many patients are entitled to financial assistance in the form of transport money and food vouchers from the Municipality of Riga, patients can only receive social support if they are declared residents, with a documented address within Riga City. This presents a challenge for homeless patients or those who are registered in different cities yet live in Riga. Dainis, for example, did not have access to a general doctor because he was not declared in Riga City, and only obtained the declaration when he spent some time in a shelter.

Applying for social assistance can be cumbersome. Ilze, a social worker based part-time at the ambulatory clinic, establishes whether a patient is entitled to financial assistance, and helps eligible patients fill out the application. She reviews the patient database to verify patients’ social and residency status, however sees few—if any—patients in person on a daily basis, instead relying on phone calls to make contact. This is challenging, she avowed, as patients were often resistant to being called and reluctant to provide information about their whereabouts or social contacts. Ultimately, the assistance provided may only make a small difference for the financially least well-off patients, and often falls short of patients’ needs. Kristine, one of the DOT nurses was vocal in her critique:

*It’s too little money that the social assistance gives for food*, *there is this system of coupons and every 10 days*, *patients get 4 coupons and each coupon is worth 1 Euro 60*. *This is meant for food but it is not sufficient … It’s about 19 Euro 20 per month* [but the patients] *need more protein*, *like cottage cheese*. *It’s not enough*!

Addressing mental health issues among patients identified as being at risk of poor adherence was often restricted to finding out if there was a family member close to the patient. Yet when we asked to what extent family members accompanied patients or acted as treatment supporters, Anita, the head TB nurse, contrasted spousal and familial relations in Latvia with those in other countries: “*In Latvia*, *we don’t have these kind of strong relations…we don’t see family members who help in the treatment period*”.

Referring someone for psychological support services was not readily accepted by patients, first because of the sensitivity of the issues and patients’ reluctance to present with ‘mental’ issues [24], and second, because of the associated costs: a visit to the psychologist or a therapist was expensive and therefore potentially out of reach for vulnerable patients. There is no resident psychologist in the TB ambulatory clinic, but TB patients are sometimes referred to a counsellor from an HIV non-governmental organisation (NGO) in the city.

Unlike HIV, there was less ‘enthusiasm’ for TB, Dr A commented, and no TB NGOs she was familiar with that might provide similar psychological help. She was aware of two mentally distressed patients who had been advised to see the psychologist at the HIV NGO, however was unsure whether they had made use of this service, as there was no follow-up. Andris and Ludmila were both offered the opportunity to consult with a psychologist based on their risk profile but declined as they didn’t feel it was necessary. Some staff suggested that patients might not be comfortable with talking to health workers. Anita reflected that “…*patients don’t like to come here and spend extra time or ask for more information*. *It’s only if I see them in the corridor*”. She added: “*In Latvia*, *our mentality is that we are not so open*…”

When asked whether Ilze, the social worker, could provide some psychological support for patients, staff seemed surprised by the question, arguing that social workers did not have the training or skills to do so. Ilze herself said she would rather leave this to a doctor, as “*patients trust the doctor more*”. She reminded us: “*In this clinic*, *treatment is of primary importance*, *and social assistance is secondary… it is more auxiliary work*”. Dr C suggested that the low status of social workers in clinical settings was not a human resource issue, but rather that “…*there is no full understanding about this work*” on the part of the authorities. She noted:

If we demand more social workers, the response would be that there are enough. There is one staff member for beds and that is enough. I think the problem is that the understanding of social work is very narrow. We see only the pension, the document work, passport, immigration registration and so on and that’s it. But to go a little further to understand what we have to do, I think this is the problem of awareness.

All staff members noted numerous challenges with vulnerable patients who had alcohol or substance use issues currently, or in the past. While there was an understanding of the social determinants of excessive alcohol use, drunkenness was frequently described as making the work of staff difficult, leading to ‘bad behaviour’, ‘rudeness’ and malingering, or ‘lying’ about not being able to come in for DOT. Dainis was made aware that mixing alcohol with the drugs was dangerous as the “*drugs are like poison*”; at times, he struggled to find a balance between his drinking habit and medication, expressing concern about having to go back into isolation if his treatment was compromised. Anita worried, too, about Viktors’ past ‘heavy drinking’ habits and the possibility of his relapse: “*…if there are some pressures from diseases or family or friends or something else*, *they can go back*”.

For patients with substance abuse issues, assistance is similarly limited by the lack of integration of health and social services, and the perception that the latter do not fall within the remit of TB care. Individuals are informed about where they can get help, if they want it. This assistance compartmentalises addiction issues as separate disease conditions requiring different types of expertise. There is only one *narcologist* who works with the municipality social services; referrals to addiction specialists otherwise require payment. Dr B, one of the TB doctors in the ambulatory clinic, expressed her professional limitations in dealing with the care and follow-up of drug users on treatment, suggesting the clinic would benefit from hiring a part-time *narcologist*, if finances permitted. The limitations in skills, resources, and capacity to address ‘social problems’ as articulated by the staff have repercussions for patients’ whose capacity to adhere to treatment is compromised by structural vulnerabilities. As seen in the following section, non-adherence to DOT is not a clear-cut pattern of missed doses; rather, it signifies lapses in the ability to take care of one’s health triggered in most cases by critical events in patients’ lives.

### 3.4 ‘*It*’*s actually work to come here and take this medication*’: Starting and stopping DOT

Following assessment of their clinical and psychosocial profile, patients are started on daily DOT either in person or via a Skype call. DOT is offered at both CTLD as well as a satellite clinic in the city centre. The DOT nurse is potentially a pivotal figure in terms of maintaining contact with patients on treatment. At both DOT sites, the nurses are responsible for monitoring treatment intake and relaying information about patients who have missed their daily doses. At the outset of treatment, communication with patients is vital to reinforce the necessity of adhering to the daily schedule of treatment. As Guna, one of the DOT nurses, pointed out: “*It’s important to remind them that they need willpower… that it’s actually work to come here and take this medication*”. However, following the initial ‘induction’ to treatment, patient contact with staff becomes more sporadic and sparse over time. Both Jevgenijs and Sergejs, another study participant, said they were not familiar with the staff and did not know their surnames, and generally avoided contact with other patients. Andris was generally satisfied with the care received but commented that the attitude of the nurse providing DOT was “*like being in the army…in her room*, *you must go by her rules*”.

Viktors resented having to come to the clinic every day: “*It’s a big demand on my time*. *I understand that taking tablets is in my interest*, *but I don’t see why I have to come here all the time*.*”* Although he praised the head TB nurse Anita’s competence and ‘loyalty’, he was more critical of other staff, who he said were too caught up with their professional guidelines–*”they only say what they are supposed to*”–rather than being flexible of patient circumstances. His main concern with DOT, despite the clinic’s flexible opening hours, was that he felt it constrained his capacity to work, and therefore to pay off his debts. He was desperate to finish treatment: “*I want them to let me out as quickly as possible … so they don’t extend my sick leave*”. The CTLD has a courier, Anna, whose role is to follow up on patients who have not shown up for their daily DOT through home visits. She receives a stack of records of people who are ‘missing’ from the nurse, which she tries to process one-by-one. Her intervention is critical for vulnerable patients struggling to stay on treatment, however home visits can be time consuming, tiring, and sometimes frustratingly ineffective.

There are challenges locating addresses and getting into buildings, and occasionally, security risks. One patient who used to come regularly had stopped coming about a month ago; in way of explanation, Anna, the courier, told us that this date coincided with the anniversary of his wife’s death which he had ‘commemorated’ meaning he had resumed drinking alcohol. Although he had been placed in social housing away from the centre of town, he had returned to an earlier abode, which Anna vividly described as an old and dilapidated building with “*broken windows and a narrow and dark staircase*”, a place she was afraid to visit. Overall, Anna was modest about what she could do, emphasising that she contributed to the team, but was limited due to her lack of “*education in medicine and professional knowledge of TB*”, reinforcing once again the widely held stance that care for TB patients was foremost the domain of clinicians.

Two cases of patients who were lost to follow up illustrate the difficulties of retaining all patients in care. Ludmila, initially positive about her experience at CTLD and optimistic about staying on treatment, became more irregular in her visits about 3 months after starting treatment. After a fight with her husband, she started drinking again and moved to another city. Anita noted attempts to contact her through her son after she was ‘lost’ to the system, to confirm her health status but he said he no longer had contact with her: it was “*too difficult to help her*”.

When we met him, Amadi had been on TB treatment for nearly three months. He took his tablets regularly, although Anita noted he had nutritional deficiencies and digestive problems linked to the medication side effects. He met with her a number of times, mainly to ask for support on immigration issues. Our question about how he was doing on treatment, however, elicited a chain of causal factors that compromised his health and well-being:

*If you need to take a tablet*, *you need to eat food*, *but there isn’t any food* […] *I need to talk to the doctor because* [taking] *the tablets is difficult without food* […] *But it is difficult for us*, *we have no money*. *The government does not care about us*. *And I have no family*, *nobody to come and help*. *So I applied already* [to go to] *England because this organisation*, *they gave me the documents to go out*. *They left it with the camp*, *but I cannot earn*. *Because I have only 1*,*139 Euro but if I need to work*, *how*? *Maybe it is about 200*, *300*, *it is not enough*. *It’s a difficult life for me with my health*.

Shortly after the interview, Amadi’s appearance became more erratic, and finally, he stopped coming to the clinic. Upon inquiry, the CTLD staff was informed by one of the nurses at the refugee camp that he had left the country before Christmas; he was then classified as lost to follow up.

The reconstruction of Ludmila and Amadi’s treatment itineraries indicate how social and structural vulnerabilities affecting some patients’ ability to stay on treatment can culminate in patients’ disengagement from the health system. In part, the difficulties of responding to these vulnerabilities are due to a broader context of health care financing challenges in Latvia. As Dr C pointed out, the overall decline in TB rates has resulted in budget cuts negatively affecting support for TB patients. Anita commented that she has had a vacancy for a TB nurse open for two years: working in TB is not appealing, and small salaries do little to dispel the stigma. Two of the nurses currently working at CTLD are over 70 years old and highly valued for their loyalty and commitment to TB work. Basic health workforce gaps may limit the possibility of patient follow-up and retention. However, it is the prioritisation of medical over social aspects of TB care and the resulting divisions in practice and responsibility that precludes the integrated, patient-centred approach that might enable patients like Ludmila and Amadi to stay in care.

## 4 Discussion and conclusion

*If we can solve the social network problems*, *we can solve the medical issues*. *In some cases*, *it’s a systematic problem*, *it’s the restriction of only having 20 minutes*, *it’s a limitation of the system*. (Anita, Head Ambulatory Nurse, CTLD)

In the setting observed, the strictly followed programmatic approach to management of TB, backed by good surveillance, diagnostic and information systems, minimal delays in treatment initiation, and available medication enables effective and timely organisation and delivery of care for the majority of patients. This is reflected in the high rates of DOT adherence in the clinic. Over the course of the pilot study, 90% of 67 patients completed treatment or were cured, 5% were lost to follow up, and just 4% of the total doses administered were missed. However, within the clinic, we observed that the emphasis on clinical management and monitoring of patients frames TB care in a way that discourages the systems responsiveness to vulnerable patients on treatment. The data from Riga provides insights on the challenges of ‘localising’ ideals of patient-centred care in a context where social and structural vulnerabilities compromising patients’ treatment pathways are seen as beyond the realm of the clinic. Staff at CTLD are well aware of risk factors that are associated with irregular adherence and poor treatment outcomes. In their care practices, they display sensitivity and understanding of ‘the social’ in patients who present with multiple, overlapping risk factors [25]. In theory, responding positively to risk factors on the psycho-social risk screening tool used in the intervention study entitled a patient to three different ‘streams’ of support: social assistance, psychological support, or specific services of a substance use specialis*t*, a *narcologist*. In practice, however, the separation of these issues as distinct encourages a view that they must be tackled separately, with financial and human resources outside of the purview of TB care, rather than in an integrated manner.

The division between the ‘medical’ and the ‘social’ in TB patients governs how different staff members communicate with patients at different phases of the patient trajectory. The initial phase of coming to terms with a serious diagnosis is a crucial point for communication with patients. However, in this setting, the hospital environment, the emphasis on technical knowledge about treatment procedures and the principle of isolation, although medically sound, may reinforce patients’ sense of uncertainty, powerlessness, and dependency. As suggested in the patient and staff interviews, they may not be receptive to information at the point when they are still coming to terms with the diagnosis. The relief expressed by patients in this study when they transferred to ambulatory care and experienced what Kaspar referred to as ‘humane care’ underlines the importance of moving towards decentralised, patient-centred approaches to DR-TB care, as is the case in a growing number of resource-poor settings.

Professional hierarchies and the division of labour between doctors, nurses, and ancillary staff further hinder an integrated and patient-centred approach to patient care. A limited view of what ‘social assistance’ is on the one hand, and an overly medicalised view of psychosocial support on the other, result in the potentially misleading impression that there are not enough resources to respond to vulnerable patients. Restrictions on the job description parameters of potentially key individuals, for example, the social worker and the courier, who both said they lacked ‘professional knowledge’ about TB, result in missed opportunities for patient-centred care that takes patients’ life experiences into account. At the same time, the medicalisation of alcohol and drug use means that the TB staff see these issues as out of their domain and only manageable within specialist services that are few in number and potentially costly for patients.

A clinical approach to assessing adherence patterns quantifies individual episodes of poor adherence as ‘missed doses’. In this paper, we suggest that ‘adherence to treatment’ should be seen as part of patients’ longer health-seeking trajectories within which individuals’ options and choices to engage with the health system may be constrained by their position within risk environments [15]. ‘Risk screening’ tools for poor adherence can be modified to move away from an emphasis of individual traits to an elucidation of underlying structural issues, as recently proposed by Bourgois and colleagues [26]. Closer attention to what is communicated to the patient (and how) at different stages of diagnosis and treatment initiation may support a more patient-centred approach to knowledge transfer and treatment literacy. Small adjustments can be made within clinic routines and communication practices to support treatment adherence of vulnerable TB patients through more dialogue and interaction at critical points along their trajectory of care: these appear to be the moment of diagnosis, transition into ambulatory care, and the ‘normalisation’ of taking daily treatment. Concurring with Lucenko and colleagues [1] who emphasise the need for additional attention and support of vulnerable patients to prevent unsuccessful treatment outcomes, we see the critical importance of integrating medical and social aspects of TB care within the clinic to encourage better treatment outcomes for vulnerable patients. However, this goes beyond compartmentalising psychiatric, alcohol, and drug addiction issues separately which may only act to further fragment care and stigmatise patients.

Recent global TB control strategy statements and action frameworks [7,27] spell out the need to address underlying social determinants and incorporate social support and protection as essential components of TB care. More recently a move toward a ‘people-centred’ model of TB care, defined as care that is “…focused on and organized around the health needs and expectations of people and communities rather than on patients or diseases” [28] has been promoted by the World Health Organization’s Regional Office for Europe [29]. This move represents an important shift in the paradigm of TB care which, as we suggested at the outset of this paper, has long been dominated by a top-down and paternalistic view of patients, failing to adequately take account of the heterogeneity and diverse circumstances of individuals who contract TB. A European Union consensus statement [30] on TB control in big cities and urban risk groups specifically recommends integrated support consisting of collaboration to promote suitable housing for homeless people; providing access to social support for all vulnerable populations; and identification of barriers and promotion of access to healthcare services [30]. Evidence from older initiatives in European cities London [31] and Barcelona [32], show that interventions providing social and other support services alongside medical care can produce good outcomes for TB patients. More recent guidance on what interventions work to improve early diagnosis of tuberculosis and treatment completion in vulnerable populations features in systematic reviews of research undertaken in low to medium incidence countries [33,34]. However, beyond policy statements and promising research, operationalising integrated and patient-centred care as routine practice within strongly centralised and medicalised systems of TB control is challenging. In order for the Latvian health system to become more responsive to the complex realities of non-conforming patients, standardised programmatic approaches for TB control must be balanced against flexibility and innovation in the organisation and delivery of care. Joint action on integrating social and medical care is urgently needed, as has been proposed in other settings like the UK [35]. At the time of our submission, approval of Lativan government legislation in towards this goal was scheduled for the end of 2018 –if enacted, this may gradually bring about the required change in the way the package of TB care for vulnerable individuals in Latvia is defined and provided.
